# Surveillance for Onchocerciasis-Associated Epilepsy and OV16 IgG4 Testing of Children 6–10 Years Old Should Be Used to Identify Areas Where Onchocerciasis Elimination Programs Need Strengthening

**DOI:** 10.3390/pathogens11030281

**Published:** 2022-02-23

**Authors:** Alfred Dusabimana, Joseph Nelson Siewe Fodjo, Michel Mandro Ndahura, Bruno P. Mmbando, Stephen Raimon Jada, Annelies Boven, Eric De Smet, Tony Ukety, Alfred K. Njamnshi, Anne Laudisoit, Steven Abrams, Robert Colebunders

**Affiliations:** 1 Global Health Institute, University of Antwerp, Doornstraat 331, 2610 Antwerp, Belgium; alfred.dusabimana@uantwerpen.be (A.D.); josephnelson.siewefodjo@uantwerpen.be (J.N.S.F.); annelies.boven@student.uantwerpen.be (A.B.); eric.de.smet@telenet.be (E.D.S.); steven.abrams@uantwerpen.be (S.A.); 2 Brain Research Africa Initiative (BRAIN), Yaoundé P.O. Box 25625, Cameroon; alfred.njamnshi@brainafrica.org; 3 Provincial Health Division Ituri, Ministry of Health, Bunia P.O. Box 57, Ituri, Democratic Republic of the Congo; michelmandro8@gmail.com; 4 National Institute for Medical Research, Tanga Centre, Tanga P.O. Box 5004, Tanzania; b.mmbando@yahoo.com; 5 Amref South Sudan, Juba P.O. Box 30125, South Sudan; stephen.jada@amref.org; 6 Centre de Recherche en Maladies Tropicales (CRMT), Bunia P.O. Box 143, Ituri, Democratic Republic of the Congo; tony.ukety@gmail.com; 7 Neuroscience Laboratory, Faculty of Medicine and Biomedical Sciences, The University of Yaoundé I, Yaoundé P.O. Box 25625, Cameroon; 8 Neurology Department, Yaoundé Central Hospital, Yaoundé P.O. Box 25625, Cameroon; 9 EcoHealth Alliance, 520 8th Ave Ste. 1200, New York, NY 10018, USA; laudisoit@ecohealthalliance.org; 10 Evolutionary Ecology Group (EVECO), University of Antwerp, Universiteitsplein 1, 2610 Antwerp, Belgium; 11 Interuniversity Institute for Biostatistics and statistical Bioinformatics, Data Science Institute, UHasselt, Agoralaan Building D, 3590 Diepenbeek, Belgium

**Keywords:** onchocerciasis, onchocerciasis-associated epilepsy, epilepsy prevalence, incidence, ivermectin, OV16 antibodies, Africa

## Abstract

To eliminate onchocerciasis-associated morbidity, it is important to identify areas where there is still high ongoing *Onchocerca volvulus* transmission. Between 2015 and 2021, door-to-door surveys were conducted in onchocerciasis-endemic villages in Cameroon, the Democratic Republic of Congo (DRC), Nigeria, South Sudan, and Tanzania to determine epilepsy prevalence and incidence, type of epilepsy and ivermectin therapeutic coverage. Moreover, children aged between six and 10 years were tested for anti-*Onchocerca* antibodies using the Ov16 IgG4 rapid diagnostic test (RDT). A mixed-effect binary logistic regression analysis was used to assess significantly associated variables of Ov16 antibody seroprevalence. A high prevalence and incidence of epilepsy was found to be associated with a high Ov16 antibody seroprevalence among 6–10-year-old children, except in the Logo health zone, DRC. The low Ov16 antibody seroprevalence among young children in the Logo health zone, despite a high prevalence of epilepsy, may be explained by a recent decrease in *O. volvulus* transmission because of a decline in the *Simulium* vector population as a result of deforestation. In the Central African Republic, a new focus of *O. volvulus* transmission was detected based on the high Ov16 IgG4 seropositivity among children and the detecting of nodding syndrome cases, a phenotypic form of onchocerciasis-associated epilepsy (OAE). In conclusion, Ov16 IgG4 RDT testing of 6–10-year-old children is a cheap and rapid method to determine the level of ongoing *O. volvulus* transmission and to assess, together with surveillance for OAE, the performance of onchocerciasis elimination programs.

## 1. Introduction

Onchocerciasis, commonly known as river blindness, is caused by the filarial worm *Onchocerca volvulus* (*O. volvulus*) [1]. It is estimated that 99% of the 20.9 million *O. volvulus* infected individuals live in 31 African countries [2]. Over 70% (14.6 million) of the *O. volvulus* infected individuals are considered to have onchocerciasis-induced skin disease and 5.5% (1.15 million) to have vision loss [3]. Moreover, accumulating evidence suggest that *O. volvulus* infection is also able to trigger epilepsy in a manner that is dependent on the microfilarial (mf) load in the skin [4,5,6], so-called onchocerciasis-associated epilepsy (OAE) [7]. 

Onchocerciasis-elimination programs rely on community-directed treatment with ivermectin (CDTI) and vector control [3]. Using CDTI, the African Programme for Onchocerciasis Control (APOC) has successfully eliminated onchocerciasis as a public health problem in several African countries [3,8]. However, in some onchocerciasis-endemic areas in Africa there is still high ongoing *O. volvulus* transmission and a high prevalence of onchocerciasis-associated morbidity including OAE due to low CDTI coverage and in some areas resulting from CDTI interruptions during the periods of insecurity [7,9,10]. 

Several new promising drugs for the treatment of onchocerciasis are being tested in clinical trials [11,12], of which moxidectin was shown to reduce and maintain low skin microfilarial density for longer than ivermectin [13]. Macrofilaricides, currently only in an early phase of development, will be needed to drastically reduce the elimination time of onchocerciasis [11,12]. However, today none of these new drugs are available for mass drug administration programmes.

The interruption of *O. volvulus* transmission is evaluated by screening pooled blackflies using the O-150 PCR technique targeting parasite-specific markers and by dissecting the heads and thorax of blackflies to determine the level of infective *O. volvulus* larvae (L3 stage) under a binocular microscope [14]. Moreover, the prevalence of anti-Ov16 immunoglobulin G4 (IgG4) antibodies in children aged <10 years, determined by an Ov16 ELISA test, is also used to assess *O. volvulus* transmission interruption [14]. This method has been used by the South American onchocerciasis elimination programme to document the elimination of onchocerciasis in several Latin American countries [15,16,17,18,19], and also in some African countries such as Senegal [20] and Uganda [21]. However the threshold required to determine when it is safe to stop CDTI and to declare interruption of transmission is still under debate [22]. According to World Health Organization (WHO) guidelines, 2000 children under 10 years of age have to be tested for Ov16 antibodies, and a seroprevalence below 0.1% is required to assume a sufficient reduction of *O. volvulus* transmission such that CDTI can be stopped [14]. A modelling study suggested that the Ov16 antibody prevalence in children aged 5–14 years would perform better in predicting elimination and that a threshold value for this age group of 2.0% and even higher threshold values would be safe to use in lower-endemic areas [23]. 

While it is important to know when a CDTI program can be stopped, it is also important to identify CDTI programs that are working sub-optimally in order to strengthen them. To do so, CDTI coverage is assessed and skin snip testing has been used to monitor community microfilarial loads. There are, however, problems with both methods: CDTI coverage data reported by the community-directed distributors of ivermectin are often not very reliable [24]. Independent surveys, as recommended by the WHO, provide more reliable results but are relatively costly. Skin snip testing is also problematic because it requires punches that are difficult to obtain and that are quite expensive. It also requires an experienced lab technician and a good microscope to read the skin snips and differentiate *O. volvulus* microfilariae from other filarial larvae, with results only made available the next day. Moreover, as it is an invasive and slightly painful procedure, therefore populations are increasingly reluctant to be skin snip tested. Therefore, we propose to use the Ov16 IgG4 rapid diagnostic testing (Ov16 RDT) of children 6–10 years old as an easier alternative way to determine the degree of ongoing onchocerciasis transmission. In different onchocerciasis-endemic foci in sub-Saharan Africa, we investigated how the Ov16 RDT seroprevalence among 6–10-year-old children as a proxy for ongoing *O. volvulus* transmission, together with epilepsy prevalence and ivermectin coverage data, can be used to evaluate the performance of the onchocerciasis-elimination programs.

## 2. Materials and Methods

### 2.1. Epilepsy Surveys in Different Onchocerciasis Endemic Foci

**Figure 1:**
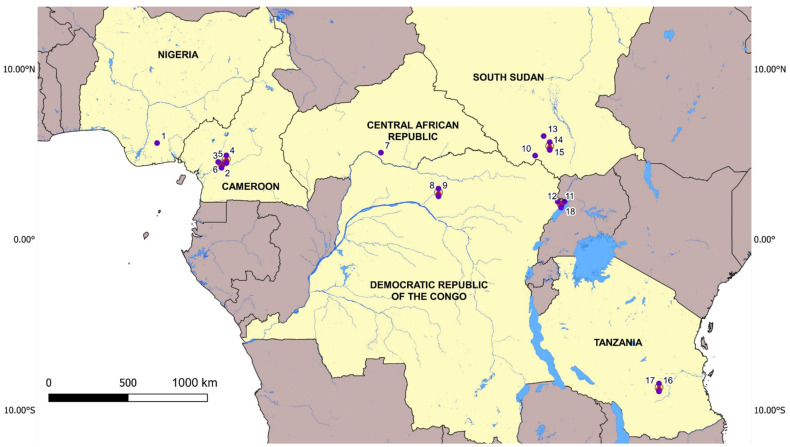
Map with the localisation of the villages included in the study; (1) Imo River Valley (Imo State); (2) Kelleng (Littoral Region); (3) Bayomen (Mbam River Valley); (4) Nyamongo (Mbam River Valley); (5) Bilomo (Centre region); (6) Ngongol (Mbam River Valley); (7) Kodjo (Landja Mboko District); (8) Makoko (Bas Uélé Province); (9) Wela (Bas Uélé Province); (10) Maridi (Western Equatoria state); (11) Kuda valley (Ituri Province); (12) Draju (Ituri Province); (13) Mvolo (Western Equatoria state); (14) Mundri center Payam (Western Equatoria state); (15) Amadi Payam (Western Equatoria state); (16) Mahenge Sub-urban villages (Ulanga district); (17) Mahenge Rural villages (Ulanga district); (18) Kuda valley (Ituri Province).

Between 2015 and 2021, door-to-door epilepsy surveys were conducted in onchocerciasis-endemic villages across Central African countries: Cameroon (Sanaga valley in the Littoral region: Kelleng [25], and Mbam valley in the Littoral region: Bilomo, Bayomen, Nyamongo, and Ngongol [25,26]); the Central African Republic (CAR) [27,28]; West African countries: Nigeria (Imo River Basin) [29]; Central and East African countries including the Democratic Republic of Congo (DRC) in Aketi, Bas Uéle [30], and Logo, Ituri [31,32]; Tanzania (Mahenge) [33]; South Sudan, Maridi [9], Mvolo [10] and Mundri, West County [34]. In total, eighteen study sites in eight different onchocerciasis foci were included in the study (Figure 1).

Two steps were used to identify people suspected to have epilepsy. In the first step, trained research assistants accompanied by village volunteers (usually community health workers) carried out house-to-house visits in the study villages. After they obtained informed consent, household members were interviewed using a validated questionnaire containing five epilepsy screening questions [35]; this questionnaire was translated into the local languages of each study site. If the answer to one of the five questions was positive, the person was suspected to have epilepsy. Additionally during the survey, household members were asked whether they had taken ivermectin during the most recent CDTI round. 

In a second step, all suspected epilepsy cases were seen by a clinical officer or medical doctor trained to diagnose epilepsy, and/or a neurologist. These clinicians took a detailed medical history of the suspected epilepsy cases and performed a complete clinical examination, and a targeted neurological evaluation to confirm or reject the diagnosis of epilepsy using a structured pre-tested questionnaire. 

Epilepsy was defined as recommended by the International League Against Epilepsy (ILAE): the occurrence of at least two unprovoked seizures with a minimum of 24 h between the two episodes [36].

Onchocerciasis-associated epilepsy (OAE) was defined using previously published criteria [7], which included: residence in the village for at least three years, the onset of epilepsy between the age of three and 18 years, the high prevalence of epilepsy in the village, normal psychomotor development prior to the onset of seizures, and no obvious cause for epilepsy obtainable from the medical history. As potential “obvious causes for epilepsy,” we considered a history of perinatal trauma (including prolonged labor and birth by emergency caesarean section), severe measles, severe malaria, encephalitis or meningitis, or head injury with loss of consciousness within the two years preceding the onset of seizures.

### 2.2. Assessment of the Level of Onchocerciasis Transmission

We assessed the degree of onchocerciasis transmission in the participating villages by testing children 6–10 years old for onchocerciasis antibodies using the Ov16 IgG4 RDT (Standard Diagnostics, Inc., Giheung-gu, Yongin-si, Gyeonggi-do, Korea). Six and 10-year-old children were only tested at certain study sites but seven to nine year-old children were tested at all study sites. After informed consent was obtained from the parents of the children, all procedures were followed as per the manufacturer’s instructions, and Ov16 RDT results were noted for each participant. Parents of the children were also asked whether their children had taken ivermectin during the most recent CDTI. In four study sites (Aketi and Logo health zones in the DRC; Mahenge in Tanzania; and Maridi in South Sudan), microfilarial loads in skin snips of persons with epilepsy were measured before the ivermectin intake.

### 2.3. Data Analysis

Categorical variables were summarized as absolute frequencies and percentages. Epilepsy incidence and prevalence, Ov16 seropositivity among children 6–10 years old, and ivermectin coverage among the different onchocerciasis foci were calculated per study site. Epilepsy incidence was estimated retrospectively by summing up all the confirmed cases of epilepsy that reported an onset of seizure within the last five years (i.e., duration of epilepsy between zero and five years) divided by the total number of individuals involved in the house-to-house survey, and dividing by five for yearly incidence. Ivermectin coverage in the population was calculated as the number of the survey participants that took ivermectin over the total number of individuals involved in the survey. The epilepsy prevalence, incidence and OV16 seropositivity with its corresponding exact Clopper-Pearson confidence intervals were visually presented. A generalized linear mixed model (GLMM) using a logit link was fitted to assess factors associated with Ov16 seropositivity (binary response) among the children residing in onchocerciasis areas with the study site being considered as the random effect to account for the correlation that can occur among children residing in the same study site. We first fitted the model to assess an association between Ov16 seropositivity and the children’s characteristics such as age and gender of the children and ivermectin intake during the last CDTI round. Secondarily, we fitted the model to investigate whether Ov16 seropositivity of 6–10 year old children could be used to assess the performance of an onchocerciasis elimination program at the population level. At the population level, the fixed variables included ivermectin coverage and epilepsy prevalence in the study site community. The GLMM results were reported as adjusted odds ratios with 95% confidence intervals (CIs). A two-sided 5% significance level was used. Data were analyzed using SAS software version 9.4, (SAS Institute, Inc., Cary, NC, USA) and R software version 4.1.2 (R Foundation for Statistical Computing, Vienna, Austria).

## 3. Results

Summary statistics related to the epilepsy surveys and the Ov16 serosurveys are presented in Table 1 and Table 2, respectively. In total, 47,935 individuals from eight different onchocerciasis foci participated in the epilepsy door-to-door surveys and Ov16 RDT were performed in 1821 children aged 6–10 years. Of these tested children, 907 (49.4%) were boys, and 1059 (58.2%) had taken ivermectin during the last distribution round. 

**Figure 2:**
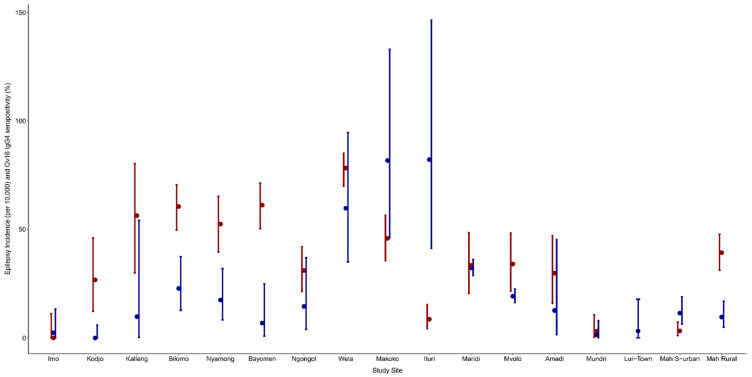
Ov16 IgG4 seropositivity (%) in children aged 7–9 years (red dots) with 95% exact Clopper-Pearson confidence interval and incidence of epilepsy (per 10,000) per study site (blue dots) with 95% exact Clopper-Pearson confidence interval in the study site.

**Figure 3:**
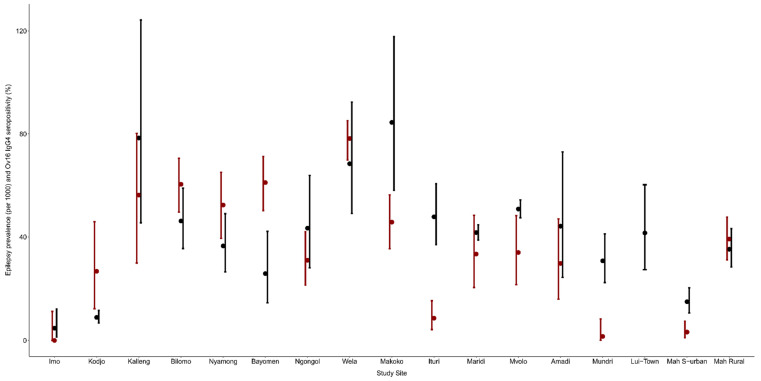
Ov16 IgG4 seropositivity (%) in children aged from 7–9 years (red dots) with 95% Clopper-Pearson (Exact) confidence interval and prevalence of epilepsy (per 1000) per study site (black dots) with 95% Clopper-Pearson confidence interval in the study site.

**Figure 4:**
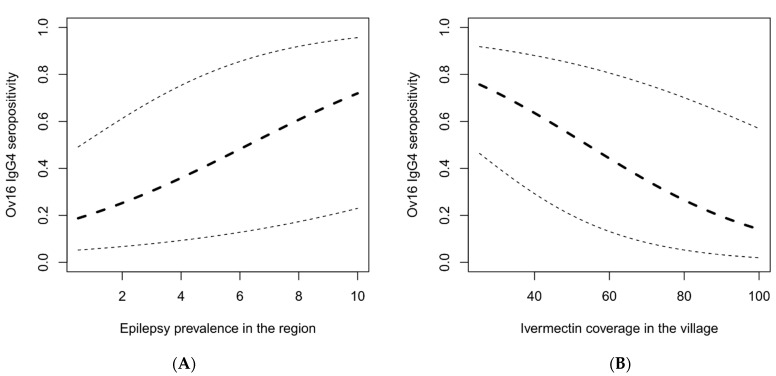
The probability of Ov16 seropositivity estimated from a generalized linear mixed model with village (excluding Ituri because of recent decrease in vector population, and excluding Kodjo in the Central African Republic because no state of the art epilepsy survey was done) considered as random effect plotted as the function of epilepsy prevalence in the study sites (**A**) and ivermectin coverage in population residing in the study site (**B**). Black solid lines represent the estimated probability of Ov16 seropositivity with pointwise 95% Wald-type confidence bands.

In all the villages, at least 75% of the epilepsy cases met the OAE criteria. However, in the Imo River Valley in Nigeria, the three persons with epilepsy meeting the OAE criteria were immigrants and had developed their first seizures in another onchocerciasis-endemic area in Nigeria. In most study sites, a high Ov16 seropositivity in children was observed in villages with high epilepsy prevalence (Figure 2 and Figure 3). A high epilepsy prevalence in the village was associated with a high Ov16 seropositivity (Odds Ratio (OR): 1.288, 95% CI: 1.194–1.390, *p* < 0.001). In contrast, a high ivermectin coverage in the village was associated with a low Ov16 seropositivity among children residing in that village (OR: 0.961, 95% CI: 0.951–0.972, *p* = < 0.001) (Table 3 and Figure 4).

The Ov16 seropositivity of six-year-old children was significantly lower compared to that of the 10 year old children; however, no difference in Ov16 seropositivity was observed when comparing 7, 8 and 9 year-old children with 10-year-old children (Table 4). 

## 4. Discussion

In onchocerciasis-endemic foci with a high epilepsy prevalence and incidence, we observed a high Ov16 seropositivity among children less than 11 years old, except in villages from the Logo health zone in Ituri, DRC where Ov16 seroprevalence was only 6.3%. In all onchocerciasis-endemic foci with a high Ov16 seroprevalence among young children, more than 75% (ranging from 75.0% to 94%) of all persons with epilepsy in the village met the criteria of OAE. In addition, a high prevalence and incidence of epilepsy was observed in areas of low ivermectin coverage, or where ivermectin was never distributed, such as in the Logo health zone. These data suggest that high ongoing *O. volvulus* transmission is associated with a high prevalence and incidence of epilepsy. 

The high epilepsy prevalence and incidence in villages in the Logo health zone, despite low Ov16 seroprevalence among young children (a proxy for ongoing *O. volvulus* transmission), is most likely the result of high *O. volvulus* transmission in the past and a recent decrease in transmission. Indeed, during a randomized clinical trial comparing the efficacy of moxidectin with ivermectin in 2009 in the Logo health zone, a high number of *O. volvulus* infected individuals with a high microfilarial load was observed [13]. However, except for one dose of ivermectin or moxidectin that was administered to the individuals who participated in this clinical trial, ivermectin and moxidectin were never distributed in this health zone. Despite a REMO assessment that had documented a prevalence of onchocerciasis nodules in Draju and certain other villages in the area, the rest of the Logo health zone had been considered to be an onchocerciasis hypo-endemic region and therefore had not been included in the CDTI program [31]. The fact that the epilepsy incidence was still high in 2016 may be explained by the fact that ivermectin-naïve children, who were already infected several years earlier, still harbored high microfilarial loads, putting them at risk of developing epilepsy even with the declining *O. volvulus* transmission. We can exclude a problem of quality of the Ov16 RDT, because at the same time the 6–10-year-old children tested negative, two persons with epilepsy meeting the OAE criteria from the same area tested Ov16 RDT positive. 

In Draju, a mountainous area located in the Logo health zone, a number of 6–10-year-old children were still Ov16 seropositive in 2016. In 2018 in the Goma and Jabi villages (which are closer to the Kuda river in the Kuda valley of the Logo health zone), only one of the Ov16 RDT tested children was positive. The explanation of the recent decline in *O. volvulus* transmission in the Logo health zone could be that the abundance of the blackfly vector of *O. volvulus* in the area recently declined possibly due to deforestation. According to the local population of the Kakoi-Koda onchocerciasis focus, they started slash-and-burn agriculture and commercial farming around 1990 which enormously increased the deforestation in this area [41]. They sometimes had to stop logging the forest because of the intensity of the blackfly bites. Local elders reported that around 1987, in the early years of the settlement of houses near the forests, the nuisance caused by the blackflies was terrible and it was sometimes necessary to flee and stay at home or to move up the hills to escape the bites [42]. However, in 2017, people mentioned that they were still being bitten by blackflies around the Kakoi river at lower altitude and during the cold season or in rainy and foggy weather [42]. A similar level of deforestation (up to 6.8% between 2000 and 2020) has also been noted in many other parts of the DRC [43,44]. Another explanation of the low *O. volvulus* transmission in the Logo health zone in DRC could be the restrictions of movements to crop fields far from houses due to the conflicts and insecurity that have increased since 2017 [45].

In a recent investigation, only two types of blackflies in the Logo area, *Simulium vorax* and *Simulium dentulosum,* were found to be infested with *O. volvulus* [46]. Of these two types of blackflies, only *S. dentulosum* was found to be infective (presence of *O. volvulus* L3 larvae in the head). *Simulium neavei* was found breeding in some rivers outside the Kakoi-Koda onchocerciasis focus. Therefore, it is possible that *S. neavei* was the main (or the only) vector in the past but recently became rare as a result of the removal of tree cover, as a result of land use changes, and because the crabs they used as substrates also became rare. In 2021 in the Goro, Jupagassa, Jupafoyo, Jupupedero and Jupumvuga villages of the Kuda valley in the Nyarambe health zone, a part of the Kakoi-Koda onchocerciasis focus where CDTI was implemented, all 7–9 year old children also tested Ov16 RDT negative. However, 90.4% of these children had taken ivermectin, but this should not have completely erased their *O. volvulus* immune response if they had been exposed. 

The highest Ov16 seropositivity among 7–10-year-old children as well as the highest epilepsy prevalence and incidence was observed in Wela and Makoko (Bas Uéle province, DRC). In these villages, CDTI had been implemented for 14 years but geographic coverage had been very low [30]. The high onchocerciasis endemicity in these villages was also shown by a REMO assessment: 43 (86%) of 50 adult men examined in Wela and 21 (70%) of 30 examined in Makoko presented onchocercal nodules [30]. While performing surveys on OAE in 2015 in the Salambongo area (Tshopo province) [32,47], *S. naevei* larvae were identified in the Aketi area on crabs in the Mobi and the Onane river [42].

A high Ov16 seropositivity among 7–10-year-old children and a high epilepsy prevalence and incidence were observed in villages in the Mbam and Sanaga valleys in Cameroon, despite many years of CDTI [25]. However, in this onchocerciasis focus, CDTI coverage has been sub-optimal. The percentage of infective blackflies in the area was found to be relatively low (0.10–0.36%), but in certain villages extremely high densities of biting blackflies were documented [48]. Despite a high Ov16 seropositivity in the children, a relatively low epilepsy prevalence (2.6%) was observed in Bayomen. This lower epilepsy prevalence in Bayomen could be explained by the high number of recent immigrants from other parts of Cameroon. Indeed, a stratified analysis including only indigenous households found a crude epilepsy prevalence of 6.7% (14/208) in Bayomen, 6.5% (15/232) in Ngongol and 5.5% (50/905) in Nyamongo [26]. The high Ov16 seropositivity in children in Bayomen concurs with the results of an entomological study that observed an even higher *O. volvulus* annual transmission based on blackfly parity and infection rates and transmission potentials in Bayomen (from dissection data) compared to Nyamongo [48].

In South Sudan, a high prevalence and incidence of epilepsy was observed in villages with high ongoing onchocerciasis transmission and with a history of many years of interrupted CDTI [9,10]. In Maridi, the highest Ov16 seropositivity and epilepsy prevalence was observed close to the Maridi dam, the only blackfly breeding site in the area where very high blackfly biting rates were observed [49]. In Mundri center Payam, the Ov16 seroprevalence was low, contrasting with a relatively high epilepsy prevalence. This is explained by the fact that the site where the children were tested was located in a more urban area further away from the river, while the epilepsy survey also included communities very close to the river.

In Tanzania, a high Ov16 seropositivity among 6–10-year-old children and a high prevalence and incidence of epilepsy were observed in the rural but not in the suburban villages of the Mahenge area [33]. *O. volvulus* transmission by *Simulium damnosum* s.l. was found to have continued in Mahenge despite 19 years of annual CDTI [50]. In 2016, the percentage of *S. damnosum* s.l. carrying infective L3 stage parasites was found to be 0.57% (95% CI 0.43–0.74%) [50], similar to infective rates reported in the 1960s [51]. In 2019, the geometric mean microfilarial density among persons with epilepsy prior to the intake of ivermectin was lowest in the Mahenge villages compared with geometric mean microfilarial densities in Maridi, Aketi and the Logo health zone [37], suggesting a lower transmission in Mahenge compared to the other sites.

In Nigeria, a 0% Ov16 seropositivity and a low epilepsy prevalence were observed after more than 20 years of CDTI in the Imo River Valley, with optimal coverage rates recorded during annual and then biannual CDTI rounds [29]. 

In Landja Mboko in the Central African Republic, an area located about 9 km from the capital city of Bangui where ivermectin was never distributed, a total of 6175 individuals were screened for epilepsy in 799 households [28]. In this study, 55 of the 75 epilepsy suspected cases examined by a neurologist were confirmed to have epilepsy, corresponding to an epilepsy prevalence of 0.89%. In addition to the 55 persons with epilepsy, five (9.1%) were classified as presenting nodding syndrome [28]. Ov16 RDT testing was performed in four settlements within the selected area at the four sites (Belespoir, Landja 1 and 2, Mangapou 2 and Kodjo), but a high Ov16 IgG4 seropositivity among 7–9-year-old children was observed only in Kodjo. Compared to other villages, Kodjo is situated only 200 m away from the Oubangui river, which most likely constitutes a suitable breeding ground for blackflies. When taken together, the high Ov16 seropositivity among 7–9-year-old children and the presence of nodding syndrome suggests that there may be a high prevalence of OAE in this area. However, because of insecurity in the area, an exhaustive house-to-house survey to assess the epilepsy prevalence was not appropriately conducted. A more in-depth investigation of the onchocerciasis and epilepsy situation in the Landja Mboko area is urgently needed to evaluate whether CDTI should be implemented in the area to prevent children from developing OAE, and to contribute to the global elimination effort.

Ov16 IgG4 seropositivity of the six-year-old children was lower compared to that among 7–10-year-old children. The reason for this lower Ov16 seropositivity among the very young children most likely resides in their different degrees of exposure to blackfly bites, as this was found to increase with age; moreover, it takes many months for exposed children to build an immune response with detectable levels of antibodies [23]. Therefore, lower titers of Ov16 IgG4 antibodies may be detected in younger children. 

Several limitations of our study need to be mentioned. To qualitatively determine the presence of onchocerciasis antibodies, only the Ov16 IgG4 RDT was used and not the Ov16 IgG4 ELISA (the gold standard technique for Ov16 IgG4 antibody detection, which is more sensitive than Ov16 IgG4 RDT) [52]. Moreover, no laboratory studies nor imaging investigations were performed to identify the causes of epilepsy. Given the cross-sectional study design, the incidence of epilepsy could only be estimated retrospectively by interview, and the information obtained could have been influenced by recall bias and the deaths of some of the individuals with epilepsy prior to the survey. We performed skin snips to determine microfilarial loads in persons with epilepsy in only four study sites; no community microfilarial load calculations were done, but we succeeded in carrying out REMO assessments in two study sites. Finally, entomological studies were carried out in only a few study sites; as a consequence we do not have data on the number of infected blackflies and biting rates in each community to determine the intensity and degree of exposure to the infected vectors per study site. 

## 5. Conclusions

Epilepsy incidence and prevalence, ivermectin coverage, and Ov16 RDT testing among 6–10-year-old children constitutes three important parameters to evaluate the performance of onchocerciasis-elimination programs and/or to identify sites where potentially such a program needs to be introduced. The Ov16 RDT, because of its low sensitivity [52], cannot be used to decide whether a CDTI program can be stopped, but could be used to rapidly assess the performance of a CDTI program in onchocerciasis-endemic areas with a high prevalence of epilepsy where no laboratory is available for performing ELISA testing and where ivermectin coverage data are not reliable. Epilepsy prevalence and incidence may also be used to estimate the performance of an onchocerciasis-elimination program. However, one needs to take into account non-onchocerciasis related causes of epilepsy and the degree of in and out migration in the area. Moreover, to assess the performance of onchocerciasis-elimination programs, other parameters such as ecological and entomological parameters also need to be considered. Our data confirm the association between high ongoing or past *O. volvulus* transmission and epilepsy prevalence. Finally, the surveys performed in the DRC (Aketi), Cameroon, and Tanzania show that many years of annual CDTI with insufficient coverage cannot interrupt onchocerciasis transmission, possibly predisposing the affected communities to a high prevalence of OAE. 

## Figures and Tables

**Table 1:** Prevalence and incidence of epilepsy, the proportion of epilepsy individuals meeting the OAE criteria, skin snip positivity of persons with epilepsy and ivermectin coverage at each study site.

Study Site, (Study Years)	Epilepsy					Ivermectin Coverage
	Prevalence	Incidence ^B^	Meeting OAE Criteria (%)	Positive Skin Snip ^D^	GMF+ (SD) ^D^	
Nigeria						
Umuoparaodu and Umuezeala, Imo river valley (2018) ^A^ [29]	4/843 (0.50%)	23.7	3/4 (75%)	0/4 (0%)		672/843 (79.7%)
Landja Mboko, Central African Republic ^C^						
Kodjo (2021) [27]	55/6175 (0.9%)		NS reported			0/6175 (0%)
Sanaga river valley, Cameroon ^E^						
Kelleng (2018) [25]	16/204 (7.8%)	98.0	93.8%			141/204 (69.2%)
Bilomo (2017) [25]	61/1321 (4.6%)	227.1	98.2%			847/1321 (64.1%)
Mbam river valley, Cameroon						
Nyamongo (2017) [26]	42/1151 (3.7%)	173.8	92.3%			
Bayomen (2017) [26]	15/582 (2.6%)	68.7	93.3%			
Ngongol (2017) [26]	24/553 (4.3%)	144.4	95.7%			
Bas Uélé, DRC						
Aketi town (2017) [30]	125/2180 (5.7%)		75.8%	18/74 (24%)	12.9 (2.1)	1219/2180 (55.9%)
Wela (2014–2016) [32]	39/570 (6.8%)	596.5				298/570 (52.3%)
Makoko (2014–2016) [32]	31/367 (8.4%)	817.4				217/367 (59.1%)
Ituri, DRC ^F^						
Draju (Logo health zone) (2016) ^G^ [31]	64/1389 (4.6%)	719.9	94.0%	66/136 (48%)	24.7 (3.2)	0/1339 (0%)
Western Equatoria state, South Sudan						
Maridi (2018) [9,37]	736/17,652 (4.4%)	321.8	85.2%	82/102 (80%)	15.0 (1.1)	7209/17,652 (40.8%)
Mvolo (2020) [10]	798/15,699 (5.1%)	191.1	78.4%			9859/13,780 (71.5%)
Mundri West County ^H^						
Amadi Payam (2021) [34]	14/317 (4.5%)	126.2	76.6%			155/317 (48.9%)
Mundri Centre Payam (2021) [34]	43/1400 (3.1%)	14.3	80.5%			775/1400 (55.4%)
Lui town Payam (2021) [34]	26/626 (4.1%)	31.9	84.0%			231/626 (37.0%)
Mahenge, Tanzania ^I^						
Sub-urban villages (2017) [33]	39/2618 (1.4%)	120.1				2039/2618 (77.9%)
Rural villages (2017) [33]	88/2499 (3.5%)	91.7	77.9%	22/42 (52.4%)	5.7 (1.6)	2028/2499 (81.6%)

^A^: Three persons with epilepsy meeting the OAE criteria were not born in the study village and had not received ivermectin prior to seizure onset. ^B^: Per 100,000 person per year. OAE: Onchocerciasis-associated epilepsy. ^C^: OAE criteria were not systematically assessed but only nodding syndrome cases were reported [28]. NS: Nodding syndrome. GMF+: Geometric mean of microfilarial load of two skin snips obtained per individuals. SD: standard error of the geometric mean. ^D^: Skin snip performed in four study sites to determine microfilarial loads in persons with epilepsy reported in Dusabimana et al. [37]. ^E^: Proportion of persons with epilepsy meeting OAE criteria were calculated based on data collected by Siewe Fodjo et al. [38]. ^F^: Persons with epilepsy meeting OAE criteria were calculated and reported by Mandro et al. [39]. ^G^: Incidence in Draju (Logo health zone) was calculated based on people with epilepsy who reported an onset of seizures within the last 12 months preceding the survey (i.e., epilepsy duration ≤ one year) divided by the total number of people who completed the questionnaire during house-to-house surveys. ^H^: Jada et al. [34]. ^I^: Persons with epilepsy meeting OAE criteria were calculated based on data collected by Bhwana et al. [40].

**Table 2:** Ov16 RDT prevalence among children 6–10 years old and ivermectin coverage among 7–9 year-old children at each study site.

Study Site (Study Years)	Ov16 RDT Seroprevalence in the Children					Ivermectin Coverage 7–9 Years
	6 Years	7 Years	8 Years	9 Years	10 Years	
Nigeria						
Umuoparaodu and Umuezeala, Imo river valley (2018) [29]	0/5 (0.0%)	0/9 (0.0%)	0/5 (0.0%)	0/17 (0.0%)	0/14 (0.0%)	21/31 (67.7%)
Landja Mboko, Central African Republic						
Kodjo (2021) [27]	2/20 (10.0%)	2/5 (40.0%)	4/12 (33.3%)	2/13 (15.4%)		0/30 (0.0%)
Sanaga river valley, Cameroon						
Kelleng (2018) [25]		3/6 (50%)	4/7 (57.1%)	2/3 (66.7%)	4/9 (44.4%)	15/16 (93.7%)
Bilomo (2017) [25]		31/52 (53.1%)	14/40 (41.7%)	10/20 (47.8%)	13/33 (39.4%)	43/112 (38.4%)
Mbam river valley, Cameroon						
Nyamongo (2017) [26]		17/32 (44.4%)	5/12 (45.5%)	11/23 (31.3%)	13/33 (42.9%)	40/67 (59.7%)
Bayomen (2017) [26]		25/39 (64.1%)	17/29 (58.6%)	13/23 (56.5%)	12/29 (41.4%)	43/91 (47.3%)
Ngongol (2017) [26]		16/36 (44.4%)	5/11 (45.4%)	5/16 (31.2%)	9/21 (42.8%)	36/63 (57.1%)
Bas Uélé, DRC						
Wela (2014–2016) [30,32]		46/60 (76.6%)	33/43 (76.7%)	18/21 (85.7%)	25/28 (89.3%)	96/124 (77.4%)
Makoko (2015–2016) [30,32]		19/43 (44%)	18/35 (51%)	6/17 (35%)	17/35 (48.6%)	90/95 (94.7%)
Ituri, DRC						
Draju (Logo health zone) (2016) [31,32]	0/51 (0%)	4/39 (10.3%)	3/41 (7.3%)	3/36 (8.3%)	2/25 (8%)	0/116 (0%)
Kuda valley (Logo health zone) (2018)	0/4 (0.0%)	0/13 (0.0%)	0/6 (0.0%)	1/11 (9.1%)	0/11 (0.0%)	0/60 (0%)
Kuda valley (Nyarambe health zone) (2021)		0/49 (0.0%)	0/19 (0.0%)	0/26 (0.0%)		85/94 (90.4%)
Equatoria State, South Sudan						
Maridi (2016) [9,37]	6/24 (25%)	11/30 (36.6%)	2/10 (20%)	3/8 (37.5%)		34/48 (70.8%)
Mvolo (2020) [10]	7/22 (31.8%)	7/15 (46.6%)	4/20 (20%)	7/18 (38.8%)		22/53 (41.5%)
Mundri West County ^A^						
Amadi Payam (2021) [34]	2/7 (28.5%)	1/11 (9.1%)	4/14 (28.6%)	6/12 (50.0%)		16/37 (43.2%)
Mundri Centre Payam (2021) [34]	1/18 (5.5%)	0/26 (0.0%)	1/23 (4.3%)	0/16 (0.0%)		18/65 (27.7%)
Mahenge, Tanzania						
Sub-urban villages (2018) [33]	0/26 (0.0%)	1/42 (2.4%)	3/65 (4.6%)	1/48 (2.1%)	5/91 (5.5%)	111/155 (71.6%)
Rural villages (2018) [33]	2/16 (12.5%)	19/52 (36.5%)	11/37 (29.7%)	26/54 (48.1%)	41/99 (41.4%)	106/143 (74.1%)

^A^: Jada et al., unpublished results [34].

**Table 3:** Generalized linear mixed model to assess an association between Ov16 IgG4 seropositivity (as a proxy for ongoing *O. volvulus* transmission in the participated community) and the ivermectin coverage and epilepsy prevalence taking into account the study village as random effect.

Effect	Estimated OR	95% CI		*p*-Value
Intercept	2.777	1.414	5.453	0.003
Ivermectin coverage in the village (in %)	0.961	0.951	0.972	<0.001
Epilepsy prevalence in village (in %)	1.288	1.194	1.390	<0.001
*Var*(*b*0) (se) ^A^	0.055 (0.170)			

*Var*(*b*0): Variance of random intercept. se: Standard error. ^A^: Ituri, DRC and Kodjo, RCA were excluded in the analysis. OR: Estimated odds ratio.

**Table 4:** Generalized linear mixed model to assess the variables associated with Ov16 IgG4 seropositivity among children 6–10 years of age, taking into account the study village as random effect.

Variables	Estimated OR	95% CI		*p*-Value
Intercept	0.439	0.201	0.959	0.040
Male gender	1.036	0.827	1.299	0.756
Female gender (reference)				
Age (6 years)	0.466	0.259	0.839	0.011
Age (7 years)	1.127	0.816	1.558	0.466
Age (8 years)	0.878	0.621	1.242	0.463
Age (9 years)	1.160	0.816	1.651	0.408
Age (10 years) (reference)				
Children ever used ivermectin	0.954	0.730	1.248	0.733
Children never used ivermectin (reference)				
*Var*(*b*0) (se) ^A^	1.551 (0.631)			

*Var*(*b*0): Variance of random intercept. se: Standard error. ^A^: Ituri, DRC and Kodjo, RCA were excluded in the analysis. OR: Estimated odds ratios.

## Data Availability

The datasets with de-identified patients’ data generated during the current study are available from the corresponding authors on reasonable request.
