# Correction: Dusabimana et al. Surveillance for Onchocerciasis-Associated Epilepsy and Ov16 IgG4 Testing of Children 6–10 Years Old Should Be Used to Identify Areas Where Onchocerciasis Elimination Programs Need Strengthening. *Pathogens* 2022, *11*, 281

**DOI:** 10.3390/pathogens11070746

**Published:** 2022-06-30

**Authors:** Alfred Dusabimana, Joseph Nelson Siewe Fodjo, Michel Mandro Ndahura, Bruno P. Mmbando, Stephen Raimon Jada, Annelies Boven, Eric De Smet, Tony Ukety, Alfred K. Njamnshi, Anne Laudisoit, Steven Abrams, Robert Colebunders

**Affiliations:** 1 Global Health Institute, University of Antwerp, Doornstraat 331, 2610 Antwerp, Belgium; alfred.dusabimana@uantwerpen.be (A.D.); josephnelson.siewefodjo@uantwerpen.be (J.N.S.F.); annelies.boven@student.uantwerpen.be (A.B.); eric.de.smet@telenet.be (E.D.S.); steven.abrams@uantwerpen.be (S.A.); 2 Brain Research Africa Initiative (BRAIN), Yaoundé P.O. Box 25625, Cameroon; alfred.njamnshi@brainafrica.org; 3 Provincial Health Division Ituri, Ministry of Health, Bunia P.O. Box 57, Ituri, Democratic Republic of the Congo; michelmandro8@gmail.com; 4 National Institute for Medical Research, Tanga Centre, Tanga P.O. Box 5004, Tanzania; b.mmbando@yahoo.com; 5 Amref South Sudan, Juba P.O. Box 30125, South Sudan; stephen.jada@amref.org; 6 Centre de Recherche en Maladies Tropicales (CRMT), Bunia P.O. Box 143, Ituri, Democratic Republic of the Congo; tony.ukety@gmail.com; 7 Neuroscience Laboratory, Faculty of Medicine and Biomedical Sciences, The University of Yaoundé I, Yaoundé P.O. Box 25625, Cameroon; 8 Neurology Department, Yaoundé Central Hospital, Yaoundé P.O. Box 25625, Cameroon; 9 EcoHealth Alliance, 520 8th Ave Ste. 1200, New York, NY 10018, USA; laudisoit@ecohealthalliance.org; 10 Evolutionary Ecology Group (EVECO), University of Antwerp, Universiteitsplein 1, 2610 Antwerp, Belgium; 11 Interuniversity Institute for Biostatistics and Statistical Bioinformatics, Data Science Institute, UHasselt, Agoralaan Building D, 3590 Diepenbeek, Belgium

There was an error in the original publication [1]. “In our multivariable analysis we first excluded the Logo health zone, Democratic Republic of Congo because of the recent decline in the *Similium* population. During the revision of our manuscript we decided to also exclude the Kodjo site, Central Africa Republic because the door-to-door survey was not well conducted due to the insecurity in the area.

During the compilation of the final version, the odds ratio after excluding Kodjo site were only updated in the Table 3 but not in the text under the results section therefore we requested the correction to update these odds ratio in the text”.

A correction has been made to “Results Section”, “Second Paragraph”:

Text before Correction:

The odds ratio reported in the following paragraph took Kodjo data into consideration. “A high epilepsy prevalence in the village was associated with a high Ov16 seropositivity (Odds Ratio (OR): 1.387, 95% CI: 1.294–1.487, *p* < 0.001). In contrast, a high ivermectin coverage in the village was associated with a low Ov16 seropositivity among children residing in that village (OR: 0.977, 95% CI: 0.969–0.984, *p* = 0.001)”.

The corrected:

“A high epilepsy prevalence in the village was associated with a high Ov16 seropositivity (Odds Ratio (OR): 1.288, 95% CI: 1.194–1.390, *p* < 0.001). In contrast, a high ivermectin coverage in the village was associated with a low Ov16 seropositivity among children residing in that village (OR: 0.961, 95% CI: 0.951–0.972, *p* = <0.001)”.

The authors apologize for any inconvenience caused and state that the scientific conclusions are unaffected. This correction was approved by the Academic Editor. The original publication has also been updated.
